# Free-Living Humans Cross Cardiovascular Disease Risk Categories Due to Daily Rhythms in Cholesterol and Triglycerides

**DOI:** 10.5334/jcr.178

**Published:** 2019-04-24

**Authors:** Azure D. Grant, Gary I. Wolf

**Affiliations:** 1 The Helen Wills Neuroscience Institute, University of California, Berkeley, US; 2 Hanze University of Applied Sciences, Groningen, NL

**Keywords:** Biological Rhythms, Circadian, Ultradian, Quantified Self, Cardiovascular Health, Cholesterol, Triglycerides

## Abstract

Cardiovascular disease risk assessment relies on single time-point measurement of risk factors. Although significant daily rhythmicity of some risk factors (e.g., blood pressure and blood glucose) suggests that carefully timed samples or biomarker timeseries could improve risk assessment, such rhythmicity in *lipid* risk factors is not well understood in free-living humans. As recent advances in at-home blood testing permit lipid data to be frequently and reliably self-collected during daily life, we hypothesized that total cholesterol, HDL-cholesterol or triglycerides would show significant time-of-day variability under everyday conditions. To address this hypothesis, we worked with data collected by 20 self-trackers during personal projects. The dataset consisted of 1,319 samples of total cholesterol, HDL-cholesterol and triglycerides, and comprised timeseries illustrating intra and inter-day variability. All individuals crossed at least one risk category in at least one output within a single day. 90% of fasted individuals (n = 12) crossed at least one risk category in one output during the morning hours alone (06:00–08:00) across days. Both individuals and the aggregated group show significant, rhythmic change by time of day in total cholesterol and triglycerides, but not HDL-cholesterol. Two individuals collected additional data sufficient to illustrate ultradian (hourly) fluctuation in triglycerides, and total cholesterol fluctuation across the menstrual cycle. Short-term variability of sufficient amplitude to affect diagnosis appears common. We conclude that cardiovascular risk assessment may be augmented via further research into the temporal dynamics of lipids. Some variability can be accounted for by a daily rhythm, but ultradian and menstrual rhythms likely contribute additional variance.

## Introduction

### Cardiovascular Disease Risk Assessment: The Utility of High Temporal Resolution Data

Cardiovascular disease (CVD) is the most common cause of death globally, making optimal CVD risk assessment a major public health priority [1,2,3,4]. CVD risk can be assessed by blood factors including low-density lipoprotein- cholesterol (LDL-c), blood pressure, glucose, total cholesterol (TC), triglycerides and high-density lipoprotein-cholesterol (HDL-c) [1,5,6,7]. These risk factors are most commonly assessed via measurement at a single time point, with the result guiding clinical action (e.g., prescription for a cholesterol-lowering statin drug) [1,8,9]. However, many risk factors vary substantially and rhythmically over the course of hours and days [10,11,12], suggesting that a single point measure may be more reliably interpreted when taken in temporal context. Indeed, accounting for short-timescale variability has improved the ability to anticipate cardiovascular events. For example, accounting for diurnal variability in blood pressure has allowed clinicians to determine that the absence of a nightly dip indicates elevated cardiovascular risk [7,11,13,14,15,16], and that individuals are more likely to experience chest pain [17] or heart attack [18] in the morning hours. Other risk factors, like blood glucose, vary significantly by time of day (e.g., Dawn phenomenon, ultradian glucose pulsatility), and current research aims to use knowledge of this variability to personalize clinical recommendations [19,20,21,22,23,24,25].

There is substantial evidence to suggest that lipids, including TC and triglycerides, have a daily rhythm in humans [26,27,28,29] and in rodent models [30,31,32,33]. In humans, laboratory-based lipidomic and metabolomic studies have illustrated circadian control of a large number of lipid blood factors, including fatty acids, triacylglycerides, glycerophospholipids, sterolipids and sphingolipids [33,34,35]. These studies specified that daily and likely *circadian* rhythmicity is present, but vary widely in experimental design, cohort, and findings. For example, in a study of young men sampled during 40 hours of wake and standardized equicaloric snacks in a hospital setting, Chua et al., found that elements of lipid-mediated energy storage, transport and signaling varied by time of day, but that phase varied up to 12 hours between individuals [36]. Separately, in a study of older adults, lipid phases agreed across subjects [37]. In a third study of 17 individuals, sampled every 4 hours for 24 hours, effects of time but not significant sine fits were observed [38]. Additionally, in 2016, a cohort of 173 observed significant change by time of day in TC by drawing blood every six hours for 24 hours in laboratory conditions [27]. At least two other human studies have demonstrated approximately 24-hour rhythms in serum cholesterol components in elderly populations in laboratory conditions, but have disagreed as to the presence of a triglyceride rhythm [39]. In 2017, a lipidomic study found diurnal oscillations in skeletal muscle lipids [40]. Additional timescales of lipid rhythmicity may be present, namely ultradian (3 h or 12 h) in rats [22,31,41,42], and menstrual [43,44] or seasonal [45] in humans.

To the authors’ knowledge, no study has focused on whether or not these fluctuations result in individuals crossing a CVD risk category, nor has this variability been quantified in free-living conditions at high temporal resolution. The putative lipid variability under such conditions is arguably the most clinically relevant, as individuals being tested for cholesterol in the clinic are likely to have variable time of day, as well as diet, exercise, sleep, and other factors prior to obtaining a clinical sample. Therefore, we aimed to assess 1) if free-living humans crossed lipid risk categories [46] on short timescales and 2) if observed variability was predictably structured within the day (i.e., showed a daily rhythm). Finally, as this project was participant-led, we aimed to 3) generate hypotheses for future study based on single-subject observations.

## Methods

### Participant-Led Research: Self-Tracking Yields Timeseries that Create Generalizable Knowledge about Biological Rhythms

The work presented here resulted from Participant-Led Research (PLR) with a general, collective goal of learning about lipid variability via high-frequency self-measurement. PLR is defined as: “An activity that characteristically aims at the socially valued goal of producing generalizable health knowledge… It is distinctive as being initiated and conducted by the participants themselves. PLR includes individuals interested in acquiring health information, whether about themselves or more generally” [47]. In contrast to a typical ambulatory study, in which all participants would collect data according to identical instructions, data in this study was collected according to each participant’s needs for a personally-designed experiment. Many such experiments involved serial within-a-day sampling, or collection of samples on several subsequent days. These data were pooled and analyzed by AG, and constitute the basis for the results presented here. Authorship was determined based on the ICJME authorship guidelines [48].

### Recruitment

People affiliated with a global self-tracking community (Quantified Self) were provided with information about the PLR either through either direct contact with the participant-organizers or via the community‘s 2017 global conference [49]. The only inclusion criteria was an interest in self-tracking cholesterol and triglycerides, and a willingness to give feedback about the organization of the project to the participant-organizers. Quantified Self Labs, a California based limited liability corporation, provided administrative support and lent testing equipment to participants.

As example of information conveyed during study recruitment follows:

*Cardiovascular disease (CVD) is the number 1 killer in the world. CVD risk is commonly assessed via annual point measurement of blood cholesterol and triglycerides. However, there is evidence to suggest that these outputs can vary significantly on short timescales. The Blood Testers project will explore whether collaborative self-tracking of cholesterol and triglycerides using a finger prick assay leads to actionable, personal knowledge*.

**Figure 1:**
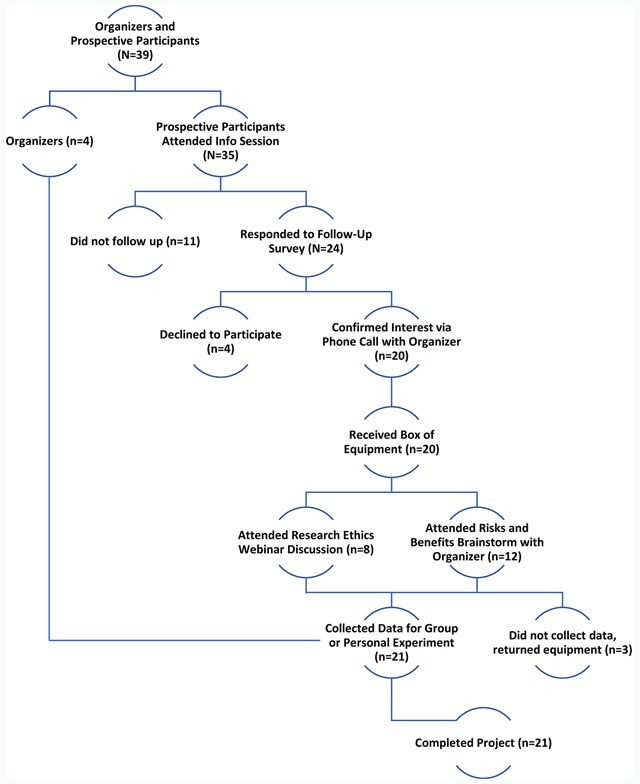
**Recruitment Flowchart.** Participant-organizers and thirty-five prospective participants met at the Quantified Self 2017 Global Conference to propose and discuss a participant-led lipid tracking project. Responders to a follow up survey confirmed their interest in participation and their goal for the project with an organizer via phone call. These individuals then received equipment from participant-organizers and attended online discussions to brainstorm risks and benefits of participation, and then to plan experiments. In total, twenty-one participants completed a project.

The above information was organized with the assistance of physicians and academic physiologists in the Quantified Self community and was additionally provided to recruits in an annotated bibliography of relevant literature. Prospective participants communicated their interest in an email or phone call with a participant-organizer. If interest persisted, potential participants filled out a survey and confirmed their participation by attending a group meeting via webcast. See Figure 1.

### Ethical Review

The study was exempt from IRB review. Nonetheless, the group felt it was important to reflect on the potential risks and benefits of participation in the project. Guidance for ethical reflection by the participants about their personal projects was provided by one of the participants who was a professor of research ethics, and who assisted in the development of a framework for ethical review of PLR based on the international WHO guidelines [40,41,42]. This expert-informed, participant-led ethical review process for creating informed consent (“self-consent“) is the subject of a separate article [70]. No intervention took place, data were aggregated and anonymized before analyses took place, and participants were not subject to any obligations to the project, excepting respectful conduct. All data were collected for personal purposes.

### Hypothesis Development

The group shared a general interest in learning the range of values lipids could cross over short periods of time, and if the structure of that variability could be predicted. It was determined that these questions could be addressed within the context of those individuals’ projects that involved high-frequency sampling.

Participants developed and investigated a hypothesis of personal interest. These hypotheses were initiated individually and developed in collaboration with other participants and participant-organizers, some of whom had academic training in circadian biology, metabolic physiology, data science, and medical practice. Topics of individual projects included morning-to-morning variability in fasting lipids, the effects of marathon training on pre-and-post run lipids, and the effects of switching to a plant-based diet on within-a-day and across-days lipid variability. See Table 1 for a complete list of group-wide and single-subject participant hypotheses.

**Table 1:** **Single Subject Hypotheses.** This table lists the hypotheses, framed by individual participants, tested in single-subject, natural experiments during the study.

Participant ID(s)	Hypothesis

1–18	Our lipids may vary significantly within-a-day.
1–12	Our lipids may vary significantly across mornings in the fasted state.
1, 17	My blood cholesterol and triglycerides may show ultradian and daily rhythms.
2	My lipids may cross a risk category within a day.
3	My post-prandial triglyceride rise may vary predictably based on the kind of food I eat.
4	My cholesterol and triglycerides may show ultradian rhythms that correlate with those in my electrogastrogram power or body temperature.
5	I can use my post-prandial triglyceride responses to create a “personal lipidemic index” comparable to a glycemic index of different foods.
6, 7	My subjectively and/or HRV-estimated stress may correlate with my cholesterol or triglyceride levels within a day.
7	Taking repeated multi-time-point “baselines” across different days may reveal stereotyped daily variability in my lipids.
8	Switching to a plant-based vegan diet may change my lipid levels within two weeks.
9	Natural variability in my lipids by time of morning may cause me to cross a risk category.
10	My daily fasting lipids, and 2-hour lipid profile may change in range or shape during very low, medium low, and moderate carb diets.
11, 14	Running may have a short term (before versus directly after a 30, 60 or 90-minute run) effect on my lipids.
11	A vegan diet may lower my total cholesterol and triglycerides over three months.
12	Tracking my lipids may be an effective encouragement for me to lose weight.
13, 16	Psychological and physical stressors (as measured subjectively and by HRV) may have distinct, measurable effects on my lipids.
15	My post-prandial triglyceride and cholesterol elevation may differ between days in which I eat three meals, and days in which I eat only one meal.
17	Changing the macro-nutrient composition of my diet for two-week increments may affect my post-prandial and daily fasted lipid levels.
19	I am interested in if my lipids and PT/INR (a measure of blood coagulation) co-vary, and if this influences the effectiveness of at home blood testing for me. Perhaps if I clot too quickly the test is ineffective.
19	I am interested in if my lipids change from before to after a) a long walk or b) a tai chi class.
20	My fasting lipids may vary predictably across my menstrual cycle.
21	I hypothesize that marathon training over two months will impact my cholesterol, and that my cholesterol may also differently from pre-to post run depending on run intensity.

### Exemplary Case Studies

All participants conducted a single-subject experiment based on one of the hypotheses listed in Table 1. Two of these single-subject experiments, described briefly below, provided interesting preliminary results relating to other timescales of biological rhythmicity potentially present in TC and triglycerides: ultradian and menstrual rhythms.

Participant 1, a 35-year-old, healthy male, took hourly triglyceride samples from 05:00 to 24:00 and recorded his perceived hunger on a four-point scale (not hungry, mildly hungry, moderately hungry, very hungry) at half-hour intervals. The participant remained at home sitting for much of the day, did no activities beyond light walking, and ate regular meals. Data were transferred from the device into a personal spreadsheet the subsequent day.

Participant 2, a 35-year-old, healthy female, took daily fasting samples between the hours of 7:00 to 9:00 on each day of her menstrual cycle. Fasting was self-reported as at least 12 hours of water only. The participant was regularly cycling and was not on birth control.

### Lipid Measurement

All lipid data was collected using FDA-approved, CLIA-waved finger-prick assay system: the CardioChek Plus (PTS Diagnostics, Indianapolis, Indiana), and Full Lipid Panel test strips (PTS Diagnostics, Indianapolis, Indiana, Lot #s 632 and 715), which directly measure TC, HDL-c and triglycerides. Participants were instructed to collect samples in the same room, under similar lighting conditions, to the best of their ability. All data and sampling conditions were self-reported by participants. Participants were strongly incentivized to report data and conditions honestly, as all samples were collected voluntarily based on the interest of that participant.

### Training

All training in data collection was delivered via: 1) video tutorial 2) live-webcast tutorial 3) one-on-one Skype coaching and/or 4) in-person training, as per manufacturer’s instructions. Each participant had access to one-on-one conversations with a participant-organizer throughout the project for any further training needed. Training efficacy was assessed first by the participant meeting or exceeding manufacturer’s standards for validity and reliability of lipid levels in a set of test samples. Second, training was considered complete if participants additionally verbally expressed readiness to move on to experimental data collection to in a meeting with a participant-organizer following the training, and prior to further data collection.

### Data Quality Validation

As a test of the reliability of both the CardioChek Plus assay and participants’ ability to self-test, all participants (n = 24) took independent sets of 2 measures, each set within 10 minutes, during which time the physiological change in blood lipids were not expected to change significantly. Three paired tests were recommended per individual, n = 50 paired tests were collected for the cohort in total. If the average of the standard deviations of the three side-by-side tests was less than or equal to the equivalent value reported by the manufacturer, participants began their personal experiments. If variability was larger than expected, participants received another training session, and all repeated the reliability test until they were able to meet the manufacturer’s standard. The standard for validity of lipid measurements is <8.9% total error (TE) [46,50]. The standard for the validity of the CardioChek Plus is slightly lower, placing an individual within the correct risk category 84–86% of trials (see CardioChek Plus online validations) and with the errant trials placing individuals within an adjacent risk category (i.e., <200 vs 200–240 mg/dL TC), for an average TE of 13%. Six participants assessed the validity of their sampling with the CardioChek Plus during a fasted visit to a physician (e.g., Labcorp, Quest Diagnostics) for a full lipid panel. Within 10 minutes of a venous blood draw, participants collected a capillary sample using the CardioChek Plus. Validations of multiple fingerstick systems including the CardioChek Plus have deemed the method comparable to venous samples [51,52,53,54].

### Within-a-Day Sampling

Most participants took multiple samples within-a-day to create a baseline curve of anticipated daily variability. Prior to serial within-a-day sampling, participants were encouraged to maintain a stable sleep and meal schedule, to the best of their ability, and to choose a day for sampling on which they did not have other obligations. On days of serial within-a-day sample collection, participants refrained from strenuous exercise, and ate according to their regular habits. Participants collected at least 4 samples within that day, at regular intervals (i.e., 06:00, 12:00, 18:00, 24:00). At the discretion of the participant, sampling frequency was increased, up to hourly, for 24 hours. For a description of sampling frequencies utilized by different participants, see **Open Science Framework** data set entitled “Sampling Statistics”.

### Repeated Morning Fasted Sampling

During 12 participants’ personal experiments, 344 total fasted morning samples were collected over a two-month period. A fasting morning sample was defined as a sample self-reported to be taken after at least 12 hours of not consuming anything except water, before 12:00 local time. Note that while there was some overlap, not all individuals who took part in within-a-day sampling took part in repeated morning fasted sampling.

### Data Management

All data collection was carried out by individual participants and recorded in a personal spreadsheet. Participants were able to either document data for private use or upload it to a group google sheet if they wished to share data within the group or publicly. Alternately, some participants opted to share their data privately with another group member for analysis without sharing to the entire group. All data analyzed in this manuscript was de-identified and shared by the participants as a public data set prior to use.

### Data Analysis

Data were collated in Microsoft Excel 2016 and Matlab 2018a. Inaccuracy (i.e., invalidity) was calculated as percent disagreement between the results of a self-collected blood test and a clinical blood test. Imprecision (i.e., unreliability) was calculated as the average percent coefficient of variation between time-paired tests taken by each individual. These representations of validity and reliability data were calculated so as to be directly comparable with the validations of the device made publicly available by the manufacturer [50]. See **Open Science Framework** for validity and reliability data. Ten days of data from ten participants were used to assess daily rhythmicity; for the purposes of sine fitting individuals with less than 6 data points within the day were excluded from the analysis. Data were converted to percent of within-a-day maximum by individual and combined in a timeseries of values by minute of sample collection. Linear interpolation using the Matlab function InterpMissing was used to generate continuous timeseries of TC and triglycerides. A two-hour moving average of this aggregate timeseries was used to represent average change by time of day. All data were plotted and analyzed in Excel 2016 or Matlab 2018a. Individual cholesterol and triglyceride data, as well as group moving averages of TC and triglycerides were fit to sums of sinewaves (of 10–12 h and 23–25 h periodicity) with 95% confidence bounds using the Trust-Region Algorithm and Non-Linear Least Squares Method within the Matlab signal processing tool box [55,56]. Periodicities and adjusted R-squared values were obtained for the auto-fit curves. Tables of supplemental statistics (RMSE, SSE, R-squared) were made available on Open Science Framework. As data were not normally distributed according to Kolmogorov-Smirnov tests, Kruskal-Wallace tests were carried out on the estimated trough regions (5:00–7:00 and 9:00–11:00) and peak regions (15:00–17:00 and 16:00–18:00) for TC and triglycerides, respectively, to assess significant change by time of day. *P* values were considered significant if <0.05. Scatter plots of individuals’ within and across day distributions were generated using the scatter function with a transparency value of 40% to illustrate frequency of overlap. Individuals were organized from low to high by median TC value, and this order was maintained across all scatter plots. All figures were formatted in Microsoft Power Point 2016 and Adobe Photoshop 2016. This manuscript conforms to the STROBE cohort reporting guidelines [57].

## Results

### Participant Demographics

The final group consisted of 24 participants, 6 women and 18 men, ages 22–70 years (median 36 years, standard deviation 12 years). Twenty-one out of 24 (88%) of participants completed the project. Participants lived in 6 countries and were of White European, Middle Eastern, or Indian descent. Sixty-one percent of interviewed participants had no formal research experience, 23% had professional (e.g., Master’s Degree or higher in a scientific field) training, but were not career researchers, and 14% were actively pursuing a research career.

### At-Home Lipid Testing in the QS Blood Testers Group Meets or Exceeds Manufacturer’s Reliability and Validity Specifications

Reliability: Average percent coefficient of variation for all time-paired tests (n = 25 paired tests) was 4.15%, comparable to that of the manufacturer’s report (4.45%).

Validity: The four participants who volunteered to take a time-paired CardioChek fingerstick and clinical full lipid panel averaged 10% +/– 3.8% TE for TC, 8% +/– 7% for HDL-c and 15% +/– 4.5% for triglycerides, and one outlier with very low triglycerides and an error of 61%. The average TE was therefore 11% +/– 5%, very close to the current gold standard. (The average TE manufacturer has reported across several large validations is 18% for TC, 8% for HDL-c and 13% triglycerides).

### Within-a-Day and Across Morning Variability Traverses Risk Categories in 90–100% of Cohort

**Figure 2:**
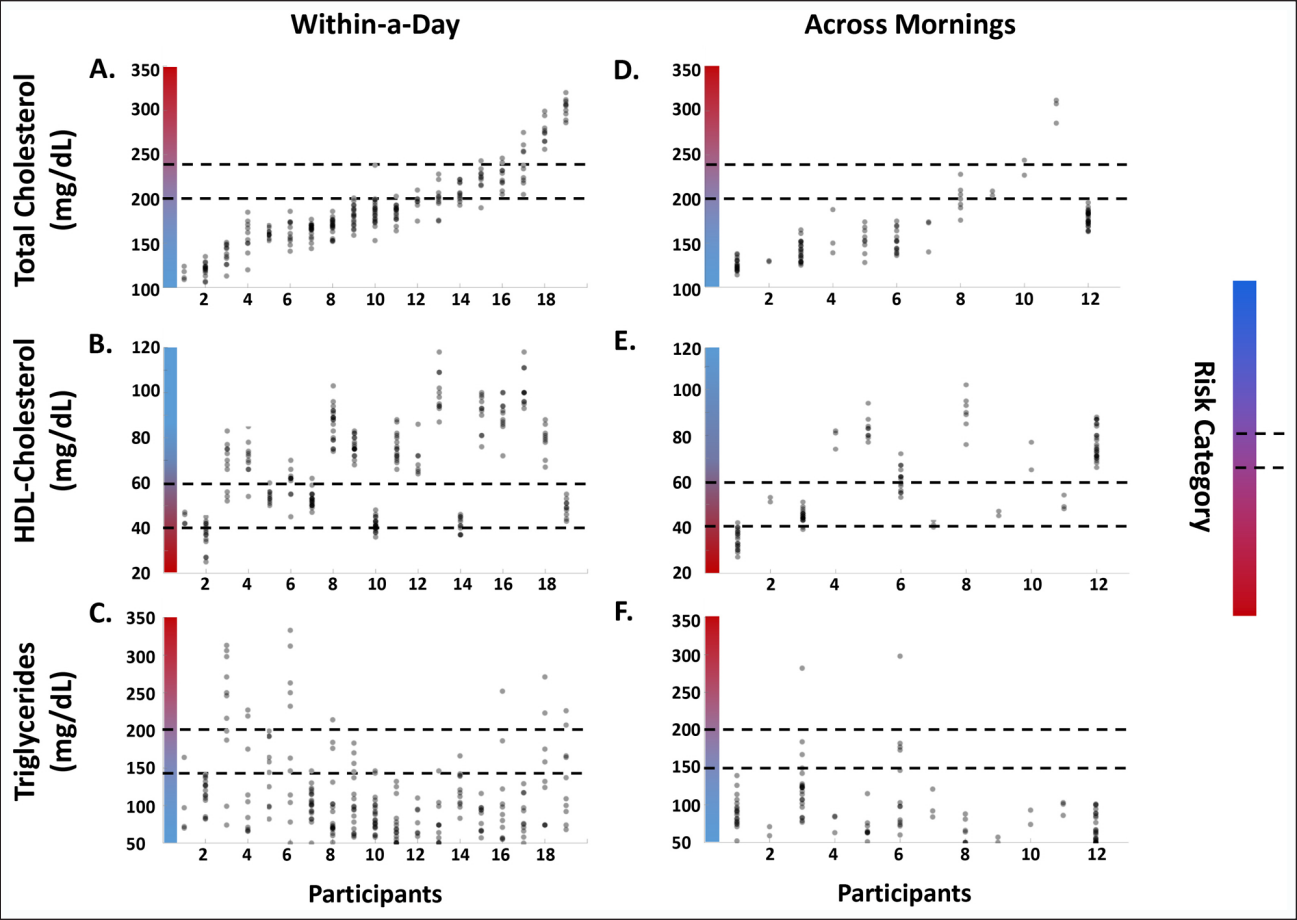
**Within and Across Day Lipid Variability Traverses CVD Risk Categories.** The daily and across morning ranges of TC, HDL-c, and triglycerides carry many individuals across CVD risk categories. Gradient indicates risk category, with redder as higher risk. Lines indicate risk category boundaries. Data shown are scatters by individual of TC, HDL-c and triglyceride values taken within a single day **(A–C)**, and fasted within a single morning on different days, between 06:00 and 08:00 **(D–F)**. Individuals are sorted from low to high range in TC, and this sorting is maintained in all plots. In total, 100% of individuals cross a risk category in as least one output at one or more time points within a day (A–C). 90% crossed at least one risk category in one output across days (D–F). Forty-seven percent of individuals cross at least one risk category in TC (A) and HDL-c (B) within a day. (C) Seventy-four percent of individuals cross at least one risk category for triglycerides. (D) Fasted between 6–8 am, 15% of individuals cross a TC risk category. (E–F) Thirty-six percent crossed an HDL-c or triglyceride risk category.

Within-a-day variability resulted in 47% of individuals crossing at least one risk category (i.e., low risk to moderate risk, or moderate risk to high risk) in TC or HDL-c (Figure 2A–B), while 74% of individuals crossed at least one risk category for triglycerides (n = 19 participants for all within-day measures) (Figure 2C). All individuals crossed at least one risk category in at least one output. Median within-a-day variability was 39 +/– 17 mg/dL in TC, 22 +/– 8.1 mg/dL for HDL-c and 59 +/– 65 mg/dL in triglycerides. High TC was weakly positively correlated with higher HDL-c in 84% of participants, but not correlated with triglycerides (data not shown).

As within-a-day measurements were not fasted after the first time point, fasted samples taken on subsequent days were assembled (n = 12 participants) to investigate if this would reduce variance. Contrary to our expectations, variability in the fasted state still resulted 90% of individuals crossing at least one risk category on separate mornings. 18% of individuals crossed a risk category in TC (Figure 2D), while 36% crossed a risk category in HDL-c or triglycerides (Figure 2E–F). Median across-mornings variability was 61 +/– 22 mg/dL for TC, 24 +/– 14 mg/dL for HDL-c, and 59 +/– 108 mg/dL for triglycerides. Higher fasted TC was not associated with higher HDL-c and was weakly associated with higher triglycerides (data not shown). Three fasted time windows were separately used to generate scatters like those in Figure 2D–F, 06:00–08:00 (Figure 2D–F), and 08:00–10:00 or 10:00–12:00. In all time windows all individuals crossed at least one risk category in one output, excepting participant 12 (data not shown for 08:00–10:00 and 10:00–12:00). The particular output(s) (TC, HDL-c or triglycerides) that traversed a risk category varied randomly by time window (e.g., a participant might cross a risk category in triglycerides between 06:00–08:00, in HDL-c between 08:00–10:00 and in TC between 10:00–12:00). There was a non-significant trend toward lower within and across day TC variability in older participants, and in the two participants who reported taking statins during, or within the last year, of the study period (data not shown). No consistent sex differences were observed, perhaps due to the combination of small sample size (n = 7) and potentially due to the wide age range of women participating (i.e., ages 22–65), which is perhaps a larger effect than any sex difference (data not shown).

### Total Cholesterol and Triglycerides Show Significant Change by Time of Day Under Free-Living Conditions

**Figure 3:**
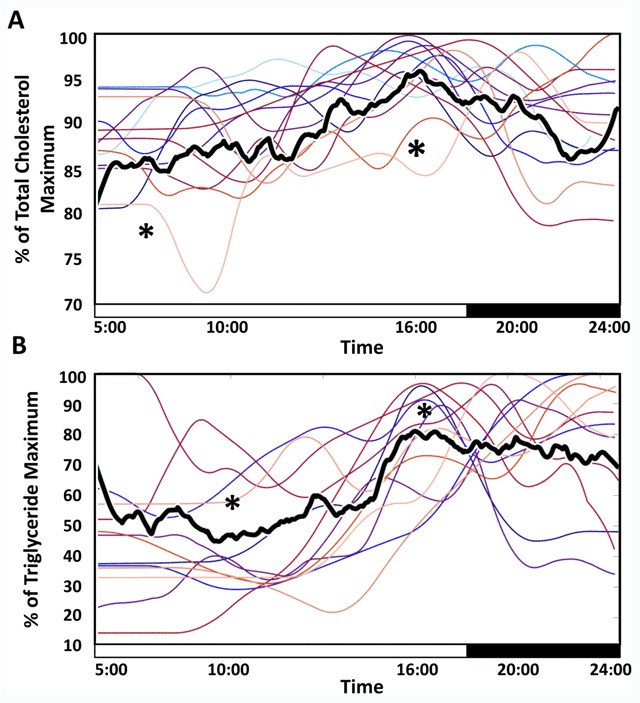
**Median Total Cholesterol and Triglycerides Show Significant Daily rhythms.** Median of all individuals’ percent of maximum TC **(A)** and triglycerides **(B)** by time of day show significant sine fit at approximately circadian periodicity, and significant change from fit peak to trough. Two-hour moving means of individual profiles are shown in color for ease of visualization. TC and triglycerides reached a maximum around 16:00, with minima in the early to mid-morning. The median percent change from maximum to minimum was 40% for TC and 35% for triglycerides. * indicate significant difference between peak and trough distributions (5:00–7:00, and 15:00–17:00 in TC and 9:00–11:00 and 16:00–18:00 in triglycerides) (Kruskal-Wallis p = 3.41*10^–41^ and p = 2.11*10^–30^). Sine fit statistics and plots of individual data and fits are available at our Open Science Foundation page.

TC and triglycerides showed a significant change by time of day (n = 13). (Figure 3A–B). HDL-c did not show a significant daily rhythm or change by time of day. Mean TC fluctuation was 40% across the day (39 mg/dL). Mean triglyceride fluctuation was 34% (126 mg/dL). The strongest sum-of-sines fit periodicities were 22 and 23 hours for TC (adjusted R^2^ = .94) and triglycerides (adjusted R^2^ = .91), respectively. Individuals’ data were individually fit to sines with periods of 23–25 h, with average adjusted R^2^ = .77 (See **Open Science Framework**). Peak and trough values were significantly different for both TC and triglycerides (p = 3.41*10^–41^ and p = 2.11*10^–30^, respectively).

### Single-Subject Experiments Suggest Total Cholesterol and Triglyceride Rhythms Occur Across Timescales

**Figure 4:**
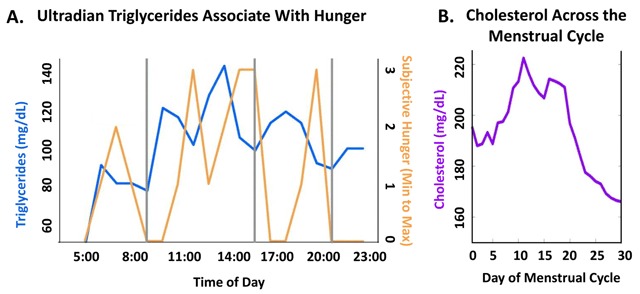
**Single-Subject Experiments Illustrate Triglyceride and Total Cholesterol Rhythms Across Timescales.** Single subject recordings of **(A)** Ultradian fluctuation of blood triglycerides inversely corresponding to subjective hunger intensity, and **(B)** TC fluctuation across the menstrual cycle captured by morning fasted recordings.

Two individuals collected data sufficient to support the hypothesis that lipids fluctuate at additional timescales: ultradian and menstrual. Each participant conducted a personal experiment of interest, providing preliminary illustrations of biological rhythms in TC and triglycerides at the ovulatory and ultradian timescales, respectively. Participant 01 recorded a strong antiphase relationship between perceived hunger and ~4-hour fluctuations in his triglycerides within a single day from wake to sleep (Figure 4A). While the participant consumed a meal after the first, third, and fourth peak in triglycerides, the second and fifth peaks maintained the antiphase relationship without the consumption of food. This may indicate an endogenous phase relationship between ultradian triglycerides and hunger not previously reported on, to the authors’ knowledge. Participant 02 reported a fluctuation of over 50 mg/dL in her morning fasted TC across her menstrual cycle (Figure 4B). Values rose during the follicular phase, coming to a peak near likely ovulation, and sharply dropping across the luteal phase.

## Discussion

This study assessed if free-living individuals crossed cholesterol and triglyceride risk categories on short timescales, and if any present variability was predictably structured within the day. All participants in our cohort crossed at least one lipid risk category both within-a-day and across fasted mornings. This variability appears non-random, as all individuals show significant daily change in TC and triglycerides, with most individuals having lower levels in the morning hours. Further, single-subject observations suggest that menstrual and/or ultradian rhythms may account for additional variability in these outputs. These findings support the idea that CVD risk assessment may be improved by deeper study of lipid dynamics across timescales (e.g., by incorporating individual’s daily rhythms into interpretation of point measurements).

This study is limited both by small sample size, and by potential confounds of everyday life not accounted for (e.g., uncontrolled meal timing). Additionally, thorough training in sample collection and personal motivation to collect quality data are not likely sufficient to completely eliminate the well-documented errors associated with citizen science [58,59,60]. Nonetheless, the within-individual measurements, high reliability of the device used, and supporting literature give us confidence that clinically-relevant lipid variability structured by time of day is present in free-living individuals.

A number of barriers stand in the way of using such self-collected data to augment scientific understanding of individuals’ cardiometabolic health. Perhaps the most important of these barriers is a lack of consensus about the legitimacy of self-collected data and Participant-Led Research. Obvious concerns include the sufficiency of participant training in data collection; these can be addressed through use of equipment approved for public use, thorough training of each participant at the study outset, and validation of data quality (see methods and results). Additionally, self-collected data reflects the inherent noisiness of everyday life (e.g., social jetlag). However, understanding the high temporal resolution dynamics of blood biomarkers in free-living humans has historically been limited by the cost of conducting clinical trials, participant willingness to undergo high frequency sampling in a clinical setting, and the slow permeation of the field of chronobiology into other domains (and hence a lack of field-specific precedent for the value of these timeseries). Although self-collected data contains the ‘noise’ inherent to everyday life, it may help generate these high temporal resolution data more quickly than would be possible through traditional methods. Further, there is likely a trade-off between slightly higher error in consumer devices and increased ease of generating timeseries data within individuals. That is, while samples collected in the course of everyday life may have greater uncertainty within each point measurement, in aggregate they may provide a more complete picture of longitudinal, within-individual physiological dynamics [61]. Such longitudinal measures capture much more than the ‘snapshot’ obtained in annual check-ups, and therefore are of interest both to the curious individual participant, and the scientific community. In this case, individuals given the opportunity to self-collect data were able to conduct the highest temporal resolution mapping of lipid variability in humans during everyday life.

Despite the limitations of PLR, the balance between personally applicable and broadly generalizable results means that participatory research may have two benefits distinct from those of traditional research. First, individuals can be motivated by their curiosity to collect more extensive data sets (and therefore provide a more complete picture of physiological dynamics) than would be possible in a traditional study. For instance, the observation of antiphase hunger and triglycerides has not been previously reported to the authors’ knowledge, perhaps due to the high frequency, within-individual sampling required to observe ultradian fluctuations. Likewise, change in morning fasted TC across the menstrual cycle has been only sparsely reported as lower temporal resolution group means (e.g., 7 samples per cycle). Some of these studies report cyclic change consistent with the participant’s [43,44,62]; others report opposite [63] or no change [64], potentially due to group averages and low-frequency sampling that would obscure within-individual patterns. Second, results of PLR are immediately and directly relevant to each individual. In this case, both individuals observed a personally-relevant pattern (e.g., knowledge that TC testing during the follicular phase might yield a higher value), simultaneously providing hypotheses for testing in a larger cohort.

The wider incorporation of PLR studies into scientific practice stands to benefit clinical practice as well. The American College of Cardiology recently released a recommendation stating that TC and HDL-c “vary little between the fasting and non-fasting state” [65], implying that variability of these outputs is not relevant for interpreting clinical results. This conflicts with the variability observed in this cohort, and those of previous studies [26,27,28,29,34,35,36], in which fasting state and/or time of day play a role. Additionally, recommendations on which biomarkers take precedence in risk evaluation vary considerably by country [1,9,66] and by year [9,46]. For instance, the current focus in the United States is on LDL-c [9], but public guidelines from the CDC [67] and NIH [68] currently list high TC as a central risk factor. Not surprisingly, a recent survey found that many physicians do not change their practices wholesale to accomodate each new set of recommendations [69], and many still rely on TC, HDL-c and triglycerides to assess risk. Reconciling observed variability with diverse recommendations and practices is undoubtedly a long-term process. Our data support the idea that self-collected, high temporal resolution measurement may contribute to this process by yielding insights relevant both to the individual and to the scientific community.

## Conclusions

This PLR illustrates that blood lipids cross cardiovascular disease risk categories on short timescales (hours to days), and that this variability appears structured in free-living individuals as a daily rhythm in TC and triglycerides. Single-subject observations support the hypothesis that ultradian and ovulatory rhythms may additionally be present in these outputs. This potentially multi-timescale variability affected clinical risk categorization in all subjects, suggesting that improved understanding of lipid dynamics would augment CVD risk evaluation. To the authors’ knowledge, this represents the highest temporal resolution data set of blood lipids collected in free-living humans. Finally, this study illustrates that individuals can use self-collected lipid data to create generalizable health knowledge and to make consequential observations about their own physiology. Future work utilizing high temporal resolution self-collected timeseries data would speed discovery in the field of biological rhythms, as well as facilitate personal learning in interested members of the public.

## Data Accessibility Statements

All data and code can be found in deidentified form on Open Science Framework by searching the title of this manuscript. Data are organized in excel files as the following: *File 1 Timeseries and Validation Data*: 1) Daily Timeseries for Each Participant. 2) Across-Morning Timeseries for Each Participant. 3) Across-Day-and-Cycle Timeseries for Single Subject Experiments. 4) Data and Statistics for Validity and Reliability of a Point-of-Care Lipid Testing Device in a Participant-Led Cohort. *File 2: Sampling Statistics by Participant*: 5) Sampling Frequency by Participant and Total Samples Collected Per Participant. *File 3: Individual Fit Statistics for Within-a-Day Profiles*: 6) Individual Sine Fit Statistics for Assessing Daily and Hourly Rhythmicity of Total Cholesterol and Triglycerides Within-a-Day. *File 4: Code for Lipid Figures*. 7) Recapitulates all analyses in the manuscript. *File 5: Individual Cholesterol and Median Fits*. 8) Shows individual total cholesterol data scatter plots overlaid with sum of sines fits. *File 6: Individual Triglyceride and Median Fits*. 9) Shows individual triglyceride data scatter plots overlaid with sum of sines fits.

## Abbreviations

TC = Total Cholesterol, HDL-c = high-density lipoprotein cholesterol, LDL-c = low density lipoprotein cholesterol, PLR = participant-led research, QS = Quantified Self.
